# Pituitary volume reduction in schizophrenia following cognitive behavioural therapy

**DOI:** 10.1016/j.schres.2017.04.035

**Published:** 2018-02

**Authors:** Preethi Premkumar, Danielle Bream, Adegboyega Sapara, Dominic Fannon, Anantha P. Anilkumar, Elizabeth Kuipers, Veena Kumari

**Affiliations:** a Department of Psychology, School of Social Sciences, Nottingham Trent University, Nottingham, UK; b Department of Psychology, Institute of Psychiatry, Psychology and Neuroscience, King's College London, London, UK; c NIHR Biomedical Research Centre for Mental Health, South London and Maudsley NHS Foundation Trust, London, UK; d South London and Maudsley NHS Foundation Trust, London, UK; e Research & Development, Sovereign Health Group, San Clemente, CA, USA

**Keywords:** Cognitive behavioural therapy, Hypothalamic pituitary adrenal axis, Memory, Stress regulation

## Abstract

Cognitive behavioural therapy (CBT) for psychosis (CBTp) aims to lower the stress of psychotic symptoms. Given that the pituitary is involved in stress regulation, CBT-led stress reduction may be accompanied by a change in pituitary volume. This study aimed to determine whether CBTp reduces pituitary volume in schizophrenia. The relation between pre-therapy memory and CBTp-led pituitary volume change was also examined given that poor memory relates to a blunted cortisol awakening response, denoting impaired stress response, in schizophrenia. Pituitary volume was measured at baseline in 40 schizophrenia or schizoaffective disorder patients and 30 healthy participants before therapy. Pituitary volume was measured again 6–9 months after patients had either received CBTp in addition to standard care (CBTp + SC, *n* = 24), or continued with standard care alone (SC, *n* = 16). CBTp + SC and SC groups were compared on pituitary volume change from baseline to follow-up. Pre-therapy memory performance (Hopkins Verbal Learning and Wechsler Memory Scale – Logical memory) was correlated with baseline-to-follow-up pituitary volume change. Pituitary volume reduced over time in CBTp + SC patients. Additionally, pre-therapy verbal learning correlated more strongly with longitudinal pituitary volume reduction in the CBTp + SC group than the SC group. To conclude, CBTp reduces pituitary volume in schizophrenia most likely by enhancing stress regulation and lowering the distress due to psychotic symptoms.

## 1 Introduction

The pituitary plays a key role in physiological stress regulation. Stress stimulates the hypothalamic-pituitary-adrenal (HPA) axis to release corticosteroids from the hypothalamus and anterior pituitary (Appiah-Kusi et al., 2015). In turn, the hypothalamus and anterior pituitary stimulate cortisol release from the adrenal glands that lowers stress reactivity. Through a negative feedback loop, high plasma cortisol lowers hypothalamus and pituitary activity. The neural diathesis-stress model of schizophrenia posits that a genetic predisposition to schizophrenia combines with an accumulation of environmental factors, including psychosocial stress, that disturb the homeostasis of the HPA axis (Pruessner et al., 2017, Walker et al., 2008). This disturbance results in HPA axis hyperactivity and elevated cortisol level that affect glucocorticoid receptors in the hippocampus and medial prefrontal cortex, and increases dopamine release and prominent psychotic symptoms.

Stress regulation is implicated in cognitive behavioural therapy for psychosis (CBTp), because CBTp reduces distress due to psychotic experiences (National Institute of Health and Clinical Excellence, 2014, NICE Clinical Guideline 178). CBTp helps patients to modify their thoughts, become aware of their reactivity to stressful situations, and think less threateningly about psychotic experiences (National Institute of Health and Clinical Excellence, 2014). CBT changes appraisal of psychosocial stress and lowers cortisol levels in patients with generalized anxiety disorder by helping patients to generate strategies to overcome anxiety (Rosnick et al., 2016). Poorer parental bonding at childhood relates to lower cortisol awakening response in patients with first-episode psychosis (Pruessner et al., 2013). Certain acute psychosocial stressors (e.g. performing cognitive tasks) result in sustained cortisol elevation in schizophrenia (Nugent et al., 2015), while other social stressors (e.g. public-speaking and job interviews) decrease cortisol levels (Bradley and Dinan, 2010, Ciufolini et al., 2014). Consequently, increased emotional reactivity to daily life stressors relates to larger pituitary volume in patients with psychosis (Habets et al., 2012). However, lower pre-CBT urinary cortisol relates to CBT-led reduction in symptom severity in people with depression (Thase et al., 1996).

Pituitary volume is reflective of HPA axis structure and function, stress and psychosis severity. Greater perceived distress from adverse life events relates to smaller pituitary volume in people who have a first or second degree relative with schizophrenia (Cullen et al., 2015). Greater pituitary volume relates to higher plasma cortisol level three years later in healthy adolescent boys (Kaess et al., 2013). Also, greater pituitary volume relates to higher nocturnal cortisol in patients with depression or bipolar disorder (Axelson et al., 1992). In schizophrenia, the pituitary enlarges at the prodromal and early stages; then, it atrophies over the chronic stage (Shah et al., 2015, Atmaca, 2014). Moreover, pituitary enlargement relates to less improvement of psychotic symptoms in early psychosis (Garner et al., 2009, Takahashi et al., 2011). Nonetheless, evidence for the relation between pituitary volume and HPA axis function in schizophrenia is scarce and contradictory.

### 1.1 The role of memory in HPA axis activity and CBTp responsiveness

Memory may be associated with stress regulation and HPA axis function. Cortisol is a glucocorticoid, which binds to glucocorticoid receptors in the hippocampus and prefrontal cortex where memory is formed and stored (Wingenfeld and Wolf, 2011). Cortisol binding to glucocorticoid receptors in the hippocampus lowers HPA axis activity (Jacobson and Sapolsky, 1991). Excessive endogenous and exogenous cortisol impairs memory in healthy people (Wingenfeld and Wolf, 2011). In patients with first-episode psychosis and children at risk for psychosis, blunted cortisol awakening response (denoting the physiological arousal due to the sleep-wake transition) relates to poorer memory (Aas et al., 2011, Cullen et al., 2014). Perhaps, poor HPA axis function can damage the hippocampus and diminish memory (Karanikas and Garyfallos, 2015, Wingenfeld and Wolf, 2011). To our knowledge, no study has investigated the association between memory and pituitary volume in schizophrenia patients. There is, however, evidence of positive associations between pre-therapy verbal memory and CBTp response (Penades et al., 2010), and larger hippocampal volume and CBTp response (Premkumar et al., 2009). Larger hippocampal volume is consistently associated with better verbal memory in schizophrenia (Antonova et al., 2004).

The aims of the present study were to determine whether CBTp reduces pituitary volume, and whether pre-therapy memory relates to CBTp-led pituitary volume reduction. Firstly, it was hypothesized that CBTp would reduce pituitary volume, because CBT helps patients to find stress regulation strategies and CBT reduces cortisol level (Rosnick et al., 2016). Secondly, it was hypothesized that better pre-therapy memory would relate to greater pituitary volume reduction in patients receiving CBTp, because good memory in patients receiving CBTp could lower stress-related cortisol level.

## 2 Methods and materials

### 2.1 Participants and design

Participants were 40 patients with a DSM-IV non-affective psychosis diagnosis (First et al., 2002) recruited from the South London and Maudsley NHS Foundation Trust; 24 patients received CBTp plus standard care (CBTp + SC), and 16 patients received standard care only (SC). Thirty healthy participants matched on age, sex and number of years in education, who reported no mental disorder history, were recruited from the same geographical area as the patients. Participants in this study have previously been examined for the neural effects of CBTp (Kumari et al., 2011). Patient inclusion criteria were: (1) a DSM-IV diagnosis of schizophrenia or schizoaffective disorder, (2) willingness to receive CBTp, (3) a stable dose of antipsychotic drugs for at least two years and their current antipsychotic drug for at least three months, (4) a score above 60 on the Positive and Negative Syndrome Scale (PANSS) (Kay et al., 1987), and (5) having at least one distressing positive symptom, i.e. scoring three or more on a PANSS positive item. In addition, CBTp + SC patients were those who were referred to and accepted for CBTp by the Psychological Interventions Clinic for Outpatients with Psychosis (PICuP) at the South London and Maudsley NHS Foundation Trust. By following opportunistic sampling, the psychiatrists in the participating psychiatric services recommended suitable patients to be allocated to the SC group. The Joint South London and Maudsley and the Institute of Psychiatry Research Ethics Committee approved the study. All participants provided written informed consent.

CBTp aims to reduce distress, depression, anxiety and hopelessness by minimizing interference arising from psychotic symptoms (Fowler et al., 1995). Therapy was delivered weekly or fortnightly (as preferred by the patient) over an average of 16 individual one-hour sessions for 6–9 months. In initial sessions, the therapist engaged the patient by forming a therapeutic relationship and focusing on the patient's needs. Standard care consisted of typical and atypical antipsychotic medication and six-monthly care plan assessment reviews delivered by a case management team, with a view to recovery. The case management team included a dedicated care coordinator who saw the patient at regular intervals, a psychiatrist and other specialists, such as a clinical psychologist and occupational therapist.

### 2.2 Clinical and memory assessments

Experienced psychiatrists (DF and APA) diagnosed the patients using the Structured Clinical Interview for DSM-IV (First et al., 2002), blind to the type of intervention patients received (CBTp + SC or SC). The psychiatrists assessed patients' symptom severity using the PANSS (Kay et al., 1987) before and after CBTp, or 6–9 months after baseline in the SC group. Trained doctoral-level researchers assessed participants' pre-therapy memory blind to the hypotheses being tested. Participants were assessed on Wechsler Memory Scale – III Logical Memory (WMS-LM, Wechsler, 1998) and Hopkins verbal learning test (HVLT, Shapiro et al., 1999). In WMS-LM, participants listened to two stories read by the examiner and recalled the stories immediately (immediate recall) and then, half an hour later (delayed recall). Immediate and delayed recall scores were calculated by scaling the raw scores to the age-related norms of the test. In HVLT, participants listened to a list of 12 words read out by the researcher three times. Participants recalled the list each time. Verbal learning was calculated as the total number of freely recalled items across the three trials.

### 2.3 Pituitary and whole brain volumetry

**Fig. 1:**
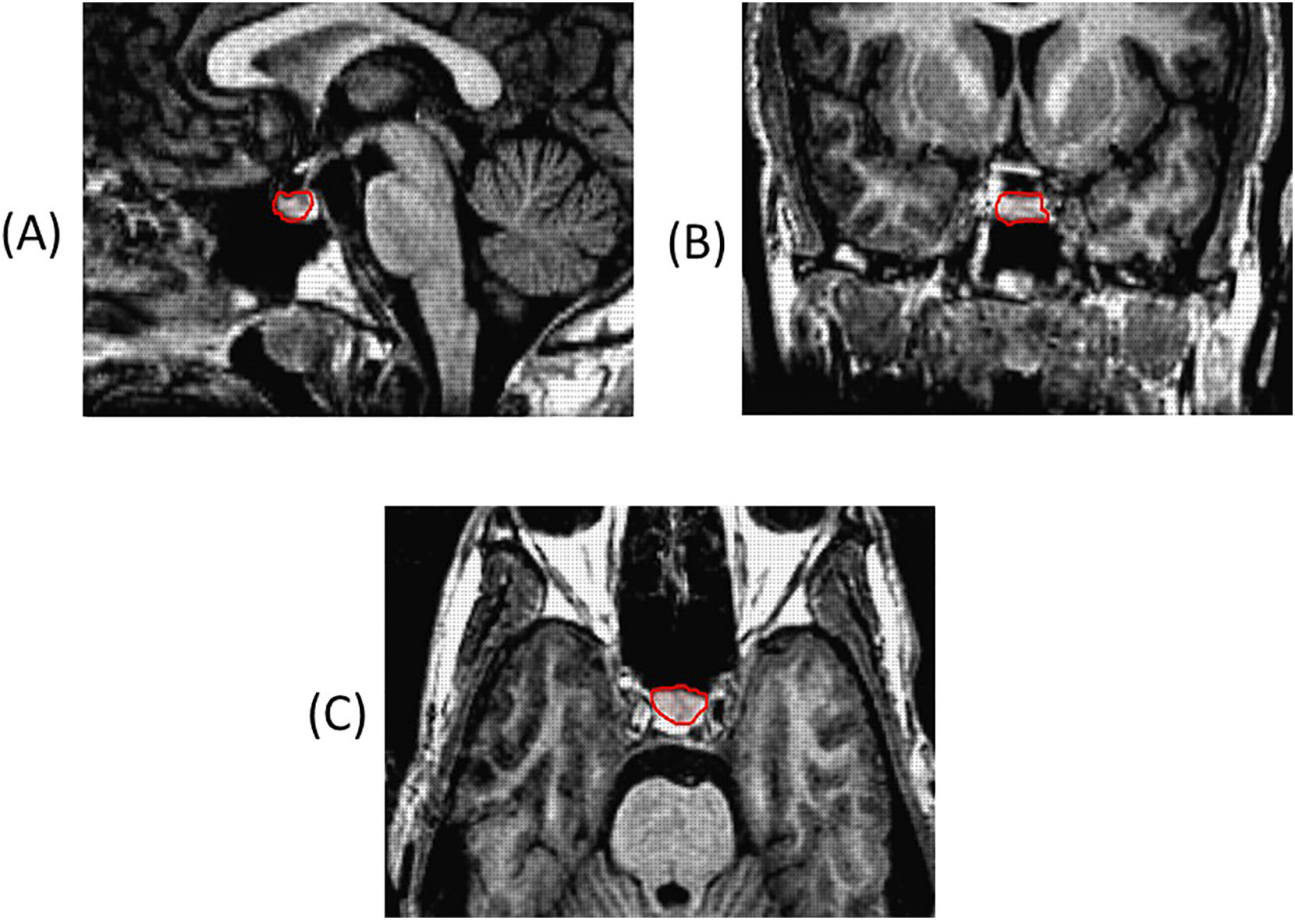
Pituitary rating (highlighted in red) as seen from the (a) sagittal, (b) coronal, and (c) axial views taken from a mid-sagittal slice.

T1-weighted structural magnetic resonance imaging brain scans were acquired in the axial plane with 1.5 mm contiguous slices from a 1.5 Tesla NV/i Signa scanner (General Electric, Milwaukee, Wisconsin) at the Centre for Neuroimaging Sciences, King's College London (TR = 18 ms, TI = 450 ms, TE = 5.1 ms, flip angle = 20° with one data average and a 256 × 256 × 128 voxel matrix). Patients were scanned at baseline and follow-up, while healthy participants were scanned at baseline only. Volumetry was performed manually using the MEASURE programme based on the Cavalieri principle (Barta et al., 1997). The Cavalieri principle states that the volume of an object may be estimated by sectioning it with a set of uniformly spaced parallel planes and measuring the cross-sectional area of the object on each plane (Barta et al., 1997). Trained researchers measured the pituitary (DB, AS and PPK) and whole brain volume (PPK) blind to group membership and treatment allocation. Novice raters achieved 95% accuracy against a trained rater's measurement of the region of interest on ten test brain scans before beginning to measure the study participants' brain scans. The pituitary was defined as a hyper-intensity adjacent to the posterior pons on the sagittal view, with clearly defined anterior and posterior boundaries (Klomp et al., 2012, Tien et al., 1992). The infundibular stalk was excluded from the segment (Fig. 1). The whole brain included the cerebral cortex, namely frontal, temporal, parietal and occipital lobes, and the sub-cortex, namely the basal ganglia and thalamus (DeLisi et al., 1995). The cerebellum, brain stem, ventricles and cerebrospinal fluid were not included in whole brain volume measurement.

### 2.4 Statistical analysis

Demographic characteristics (age, sex and years in education) were compared between groups using analysis of variance (ANOVA) or Chi-square test. Illness duration (defined as number of years since first hospitalization), change in PANSS symptom severity and change in antipsychotic dosage from baseline to follow-up were compared between patient groups. Improvement in stress-related items in the CBTp group, namely depression, excitement and anxiety, was tested using non-parametric tests (no significant symptom change in the SC group; see Results). Prolactin-enhancing drugs (all typical antipsychotics, and risperidone and amisulpride) stimulate prolactin-secreting cell proliferation, and consequently anterior pituitary enlargement (MacMaster et al., 2007, Pariante, 2008). Thus, the number of patients receiving each antipsychotic drug type, i.e. prolactin-enhancing and prolactin-sparing (all other atypical antipsychotics), was compared between patient groups. Pre-therapy memory, WMS-LM memory and HVLT scores, was compared between groups, with group (CBTp + SC, SC and healthy) as independent variable, and number of years in education and age as a covariates.

Pituitary volume at baseline was compared between CBTp + SC, SC and healthy groups using analysis of covariance, with age and whole brain volume as covariates. The longitudinal change in pituitary volume was compared between patient groups, with sex as an additional between-participants factor, and illness duration and whole brain volume at baseline as covariates. We included sex as a between-participants factor, because pituitary volume is larger in healthy women than men and in men than women with schizophrenia (Romo-Nava et al., 2013, Upadhyaya et al., 2007).

One-tailed correlations were performed to test associations between baseline-to-follow-up pituitary volume change and pre-therapy memory (scores on the HVLT and WMS-LM), because the directional hypothesis stated that better pre-therapy memory would relate to greater post-CBTp pituitary volume reduction. If a correlation was significant, a partial correlation was performed, with illness duration, whole brain volume and number of years in education as covariates. Statistical analyses were performed in Statistical Package for Social Sciences (Version 22), with *p* < 0.05 as the significance level.

## 3 Results

### 3.1 Demographic characteristics and symptom reduction following CBTp

Healthy participants were older than patients (Table 1). Specifically, healthy participants were older than SC patients (*p* = 0.04). Groups did not differ in sex, years in education, illness duration, or antipsychotic drug type at baseline or follow-up. The CBTp + SC group comprised patients with paranoid schizophrenia (79%), schizoaffective disorder (12%) and schizophrenia not otherwise specific (8%). The SC group comprised patients with paranoid schizophrenia (81%), residual schizophrenia (12%) and schizoaffective disorder (6%). From baseline to follow-up, CBTp + SC patients improved in positive symptoms, F (1,23) = 15.9, *p* < 0.01, negative symptoms, F(1,23) = 5.9, *p* = 0.02, and general psychopathology, F(1,23) = 8.6, *p* = 0.01. The SC group did not show any symptom change (*p* > 0.1). Besides, negative symptoms and overall symptoms improved more in the CBTp + SC than SC group (Table 1). When improvement in stress-related items in the CBTp group, namely depression, excitement and anxiety, was tested, depression and excitement improved significantly, z(23) = 1.96, *p* = 0.05 and z(23) = 2.29, *p* = 0.03, respectively. Improvement in anxiety approached significance, z(23) = 1.75, *p* = 0.08. Pre- to post-therapy antipsychotic dosage decreased in the CBTp + SC group, but increased in the SC group.

**Table 1:** Demographic and clinical characteristics of patients receiving CBTp + SC (*n* = 24), SC (*n* = 16) and healthy participants (*n* = 30).

Characteristic	CBTp + SC		SC		Healthy	Model	F or Chi^2^ (df)	*p* Value
Sex: men/women (*n*)	17/7		13/3		20/10	Group × sex	1.09 (2)	0.58
Age (in years)	36.1 ± 8.48		42.1 ± 9.6		34.2 ± 11.3	Group	3.3 (2,67)	0.04
Years in education	14.1 ± 3.1		13.3 ± 1.4		14.9 ± 2.1	Group	2.5 (2,67)	0.09
Duration of illness (in years)	11.4 ± 7.8		15.6 ± 12.1			Group	1.8 (1,38)	0.18
								
	Baseline	Follow-up	Baseline	Follow-up				
Prolactin-enhancing/prolactin-sparing antipsychotics at baseline (*n*)	7/17	5/19	5/11	6/10		Group × drug type at baseline	0.02 (1)	0.89
						Group × drug type at follow-up	1.3 (1)	0.25
Positive	17.8 ± 3.5	14.9 ± 3.9^a^	18.5 ± 3.7	17.1 ± 3.5	–	Group × time	1.8 (1,38)	0.18
Negative	17.4 ± 3.5	15.6 ± 4.0^a^	18.9 ± 3.7	19.9 ± 5.0	–	Group × time	4.8 (1,38)	0.03
General psychopathology	32.5 ± 5.0	28.5 ± 6.8^a^	34.9 ± 4.3	33.9 ± 7.4	–	Group × time	1.9 (1,38)	0.17
Antipsychotic dosage (CPZ equivalent)	517.3 ± 386.2	480.3 ± 385.7	503.7 ± 329.8	627.9 ± 612.5		Group × time	4.9 (1,38)	0.03
Pre-therapy memory						Group	3.2 (3,130)	0.01
WMS-LM – immediate recall	7.5 ± 2.6		6.1 ± 3.3		9.1 ± 3.0	Group	3.1 (2,65)	0.05
WMS-LM – delayed recall	8.2 ± 2.2		7.3 ± 2.5		10.0 ± 2.8	Group	3.9 (2,65)	0.03
HVLT total free recall	20.4 ± 6.4		20.9 ± 5.5		26.1 ± 4.4	Group	6.1 (2,65)	0.004

CBTp: Cognitive behavioural therapy for psychosis, CPZ: chlorpromazine, HVLT: Hopkins verbal learning test; PANSS: Positive and Negative Syndrome Scale, SC: standard care, WMS-LM: Wechsler Memory Scale – III Logical Memory test.

a CBTp-led symptom improvement.

The healthy group did not differ in WMS-LM immediate recall from the CBTp + SC group, mean difference = 1.4 ± 0.8, *p* = 0.29, or the SC group, mean difference = 2.3 ± 1.0, *p* = 0.06. The healthy group did not differ in WMS-LM delayed recall from the CBTp + SC group, mean difference = 1.5 ± 0.7, *p* = 0.1, but had better delayed recall than the SC group, mean difference = 2.8 ± 0.8, *p* = 0.05. Healthy participants had better verbal learning (HVLT free recall) than the CBTp + SC group, mean difference = 5.0 ± 1.4, *p* = 0.003, but did not differ from the SC group, mean difference = 3.3 ± 1.7, *p* = 0.2. The CBTp + SC and SC groups did not differ in either WMS-LM immediate recall, mean difference = 0.9 ± 1.0, *p* = 1.0, WMS-LM delayed recall, mean difference = 0.6 ± 0.8, *p* = 1.0, or HVLT free recall, mean difference = − 1.7 ± 1.7, *p* = 1.0.

### 3.2 Pituitary volume change from baseline to follow-up: differences between CBTp + SC and SC groups

CBTp + SC, SC and healthy groups did not differ in baseline pituitary volume (Table 2). The group-by-time interaction approached statistical significance, F(1,34) = 3.56, *p* = 0.07. Post hoc analyses compared pituitary volume at baseline and follow-up in each group individually while controlling for illness duration. The analyses showed an effect of time in the CBTp + SC group, F(1,22) = 4.54, *p* = 0.04, but not the SC group, F(1,14) = 2.97, *p* = 0.11. Antipsychotic drug type (prolactin-enhancing or prolactin-sparing) did not have an effect on pituitary volume change, i.e., the patient group-by-drug type-by-time interaction was not significant (Table 2). The main effect of time was non-significant, F(1,34) = 0.01, *p* = 0.93.

**Table 2:** Pituitary volume at baseline in healthy participants (*n* = 30), patients receiving CBTp (*n* = 24) and patients receiving standard care (*n* = 16), and at follow-up in patients receiving CBTp or standard care based on antipsychotic drug type.

Characteristic	CBTp + SC		SC		Healthy	Model	F (df)	*p* Value
	Baseline	Follow-up	Baseline	Follow-up				
Pituitary volume (mm^3^)	473.4 ± 75.9	409.1 ± 108.4	481.6 ± 132.0	445.0 ± 127.3	510.7 ± 147.9	Group difference at baseline	0.5 (2,65)	0.62
						Group × time	3.5 (1,34)	0.07
	Patients receiving prolactin-enhancing antipsychotic drugs					Group × drug type × time	0.4 (1,34)	0.5
	*N* = 7		*N* = 5					
	513.8 ± 77.9	438.2 ± 92.8	414.1 ± 85.7	392.3 ± 74.7				
	Patients receiving prolactin-sparing antipsychotic drugs							
	*N* = 17		*N* = 11					
	456.8 ± 70.8	397.1 ± 114.6	512.3 ± 41.0	469.0 ± 141.6				

CBTp: Cognitive behavioural therapy for psychosis, SC: standard care.

### 3.3 Association between pituitary volume change and pre-therapy memory in CBTp + SC patients

**Fig. 2:**
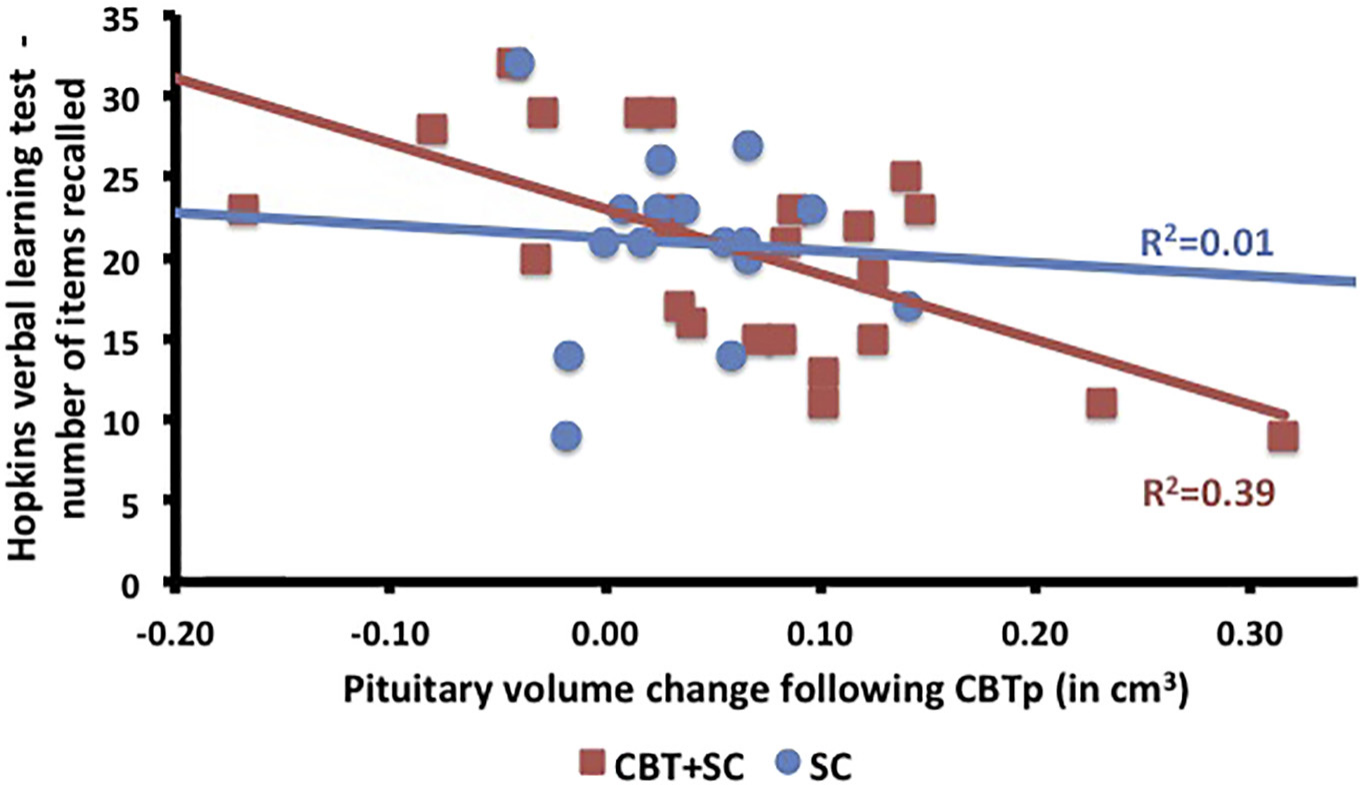
Scatterplot of pituitary volume change from baseline to follow-up against Hopkins verbal learning test score at baseline in CBTp + SC and SC patients.

In the CBTp + SC group, verbal learning at baseline was associated with pituitary volume reduction during the treatment phase (Table 3). The correlation was still significant in the CBTp + SC group after covarying for illness duration, whole brain volume and years in education, *r* = − 0.64, *p* = 0.001, but not the SC group, *r* = − 0.06, *p* = 0.419. Also, the correlation was stronger in the CBTp + SC group than the SC group as per Fisher's r-to-z transformation, z = 1.99, *p* = 0.02 (Fig. 2).

**Table 3:** Correlation, r (*p*-value), between pre-therapy memory and pituitary volume change from baseline to follow-up in the CBTp + SC (*n* = 24) and SC control (*n* = 16) groups.

	Pituitary volume	
	CBTp + SC	SC control
HVLT – free recall	**− 0.6 (0.001)**	− 0.1 (0.4)
WMS-LM immediate recall	− 0.1 (0.32)	0.1 (0.33)
WMS-LM delayed recall	− 0.1 (0.31)	− 0.05 (0.42)

Values in bold are statistically significant.

## 4 Discussion

The study aimed to examine whether pituitary volume decreases following CBTp in schizophrenia patients, as pituitary volume is a potential marker of HPA axis function. We hypothesized that pituitary volume would decrease, because CBT-led stress reduction lowers cortisol levels (Rosnick et al., 2016) and lower cortisol relates to lower pituitary volume (Axelson et al., 1992). As hypothesized, pituitary volume decreased over time in the CBTp + SC group relative to the SC group. We also hypothesized that better pre-therapy memory would relate to CBTp-led pituitary volume reduction. Better pre-therapy memory related to CBTp-led pituitary volume reduction, such that the association was stronger in the CBTp + SC group than the SC control group.

### 4.1 CBTp-led pituitary volume reduction

Our study is the first to report on the effect of CBT on pituitary volume in any disorder. Pituitary volume reduced after therapy in the CBTp + SC group. Further, the stress-related symptoms (depression and excitement) in the CBTp + SC group improved after therapy. High stress-induced plasma cortisol relates to greater severity of psychotic and negative symptoms (Tandon et al., 1991, Walder et al., 2000), whereas CBT lowers cortisol level (Rosnick et al., 2016). Thus, lower stress may reduce pituitary volume in chronic psychosis. This explanation is plausible because lower stress reactivity and lower cortisol level relate to smaller pituitary volume in patients with psychosis or bipolar disorder (Axelson et al., 1992, Habets et al., 2012). Effective stress-regulation strategies lower endogenous cortisol by improving HPA axis regulation (Abelson et al., 2014). Certain CBT stress regulation strategies may engage the HPA axis better than others. Externalizing one's stress (e.g. wanting to do good for others) decreases cortisol level more than internalizing one's stress (e.g. reducing one's appraisal of social threat) when exposed to psychosocial stress, such as a job interview (Abelson et al., 2014). CBTp may help patients to think flexibly about their psychotic experiences and perceive less social stress (Garety et al., 1997, Mason et al., 2016).

### 4.2 Relation between better pre-therapy memory and greater CBTp-led pituitary volume reduction

We found that better pre-therapy memory, specifically verbal learning, related to CBTp-led pituitary volume reduction. Greater pre-therapy verbal learning could relate to greater post-therapy pituitary volume reduction because memory activates the hippocampus. Memory-led hippocampal activation inhibits HPA axis activity (Naughton et al., 2014, Phillips et al., 2006). Furthermore, cortisol binds to glucocorticoid receptors that are abundant in hippocampus and prefrontal cortex. Cortisol binding to the hippocampus and pituitary inhibits HPA axis function (Jacobson and Sapolsky, 1991). In patients with first-episode psychosis and children at risk for psychosis, poor HPA axis function due to blunted cortisol awakening response relates to poorer memory (Aas et al., 2011, Cullen et al., 2014). Preserved memory and cognitive flexibility may enhance CBTp responsiveness in schizophrenia (Penades et al., 2010, Garety et al., 1997). Previously, we failed to find an association between pre-therapy memory and CBTp responsiveness in the CBTp + SC patients who participated in the current study (Premkumar et al., 2011). In that study however, pre-therapy memory was examined in relation to the change in composite subscale scores, rather than change in stress-related items. Also, atypical anti-psychotics may reduce elevated cortisol (Cohrs et al., 2006), and CBTp could enhance adherence to the treatment. Thus, lower stress may promote cortisol binding to the hippocampus and prefrontal cortex, and improve HPA axis negative feedback loop function.

The study has limitations. Firstly, pituitary volume was not measured in healthy participants at follow-up. Thus, it is not possible to rule out the effect of time and other non-specific effects on pituitary volume reduction. Prolactin-sparing antipsychotic drugs reduce pituitary volume more than prolactin-enhancing antipsychotic drugs in chronic schizophrenia (Klomp et al., 2012, Takahashi et al., 2012). However, drug type did not explain pituitary volume reduction in the current study. Furthermore, we included patients who were on stable antipsychotics for two years and receiving the current antipsychotic for six months. Thus, any antipsychotic medication-induced pituitary change would have been present before CBTp. Secondly, patients had normal pre-therapy pituitary volume. Normal pituitary volume in chronic schizophrenia is a consistent finding (Tournikioti et al., 2007). The apparent ‘normal’ pituitary volume in patients may be confounded by stress-related enlargement due to prolactin-secreting cell proliferation caused by prolactin-enhancing antipsychotics (Pariante, 2008). Thirdly, the small sample size of the participant groups could have increased the likelihood of a Type II error. Lastly, the lack of data on cortisol level to test HPA axis function makes it difficult to verify the functional significance of pituitary volume reduction.

In conclusion, CBTp-led symptom improvement may longitudinally lead to pituitary volume reduction. Better verbal learning may enhance recall of CBTp stress-regulation techniques, which is a potential mechanism by which pituitary volume reduction occurs.

## Conflict of interest

None.

## Contributors

Preethi Premkumar wrote the first draft of the paper and contributed to study design, data collection, analysis and interpretation. Dominic Fannon and Anantha P. Anilkumar performed diagnostic interviews and clinical assessments of patients and critically reviewed the manuscript. Danielle Bream and Adegboyega Sapara performed manual ratings of the brain regions of interest and contributed to the critical review of the manuscript. Elizabeth Kuipers contributed to the study design and critically reviewed the manuscript. Veena Kumari developed the study, raised funding, and contributed substantially to analysis and interpretation and the writing of the manuscript. All authors have given final approval of the manuscript.

## Funding

Funding for this study was provided by the Wellcome Trust, UK [067427/z/02/z, Wellcome Senior Fellowship to VK]. The Wellcome Trust had no further role in study design; in the collection, analysis and interpretation of data; in the writing of the report; and in the decision to submit the paper for publication.
